# The Consequences of Random Sequential Adsorption for the Precursor Packing and Growth-Per-Cycle of Atomic Layer Deposition Processes

**DOI:** 10.1021/acs.jpclett.4c01632

**Published:** 2024-07-16

**Authors:** I. Tezsevin, J. H. Deijkers, M. J. M. Merkx, W. M. M. Kessels, T. E. Sandoval, A. J. M. Mackus

**Affiliations:** † Department of Applied Physics and Science Education, Eindhoven University of Technology, P.O. Box 513, 5600 MB Eindhoven, The Netherlands; ‡ Department of Chemical and Environmental Engineering, Universidad Técnica Federico Santa María, 2340000 Santiago, Chile

## Abstract

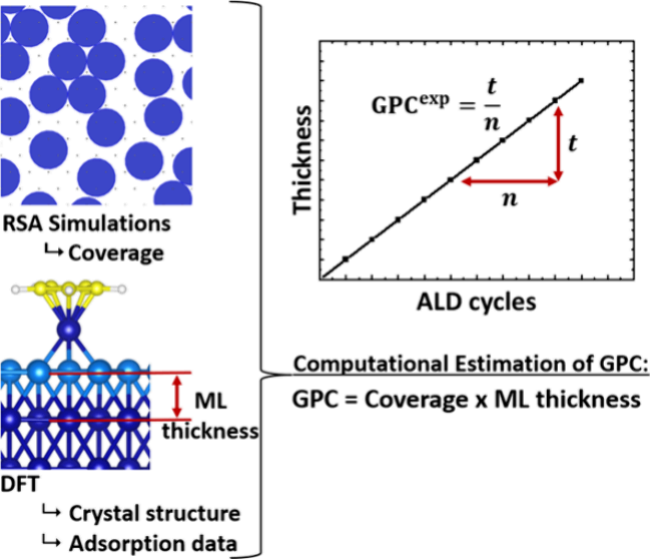

Atomic layer deposition (ALD) processes are known to deposit submonolayers of material per cycle, primarily attributed to steric hindrance and a limited number of surface sites. However, an often-overlooked factor is the random sequential adsorption (RSA) mechanism, where precursor molecules arrive one-by-one and adsorb at random surface sites. Consequently, the saturation coverage of precursors significantly deviates from ideal closed packing. In this study, we investigated the influence of RSA on precursor adsorption saturation and, consequently, on the growth per cycle (GPC) of the ALD processes. Our simulations revealed that the RSA model leads to a 22% to 40% lower surface density compared to the reference case of ordered packing. Furthermore, based on the precursor shape and size, we estimated GPC values with an average accuracy of 0.05 Å relative to experimental literature data. This work shows the critical role of RSA in ALD, emphasizing the need to consider this mechanism for a more accurate process design and optimization.

Atomic layer deposition (ALD) is a thin film deposition technique that enables the formation of uniform and conformal films of materials on various substrates. In a typical ALD process, the precursor and reactant gases are dosed on a substrate in an alternating manner separated by purge steps. ALD strictly relies on the reaction of these molecules on the substrate in a self-limiting way.^1−3^ Thus, the film thickness can be accurately controlled by the number of cycles. ALD also offers superior performance over other thin film deposition techniques in terms of property tunability.^4,5^ Based on its unique capabilities, ALD is widely used in different fields, such as nanoelectronics, photovoltaics, displays, energy storage, catalysis, and biomedical devices.^6−8^

A common misconception about ALD is that it produces a complete monolayer (ML) in each cycle, as the name seems to suggest. However, previous works clearly demonstrated that most ALD processes deposit less than a ML of material in each cycle.^4,9^ The selection of the precursor molecules is crucial for ALD, as the maximum achievable growth per cycle (GPC) depends predominantly on the precursor chemistry and structure.^10,11^ The GPC of an ALD process is generally limited either through the steric effects due to precursor size or by the chemistry between the substrate and precursors as a result of the limited availability of adsorption sites.^5,11−13^ Previous studies have modeled the GPC of ALD processes considering the close packing of precursor molecules and their steric hindrance.^14−18^ However, it should also be considered that ALD precursors are dosed in the vapor phase, such that these molecules arrive one-by-one on random surface sites.^19−22^ Consequently, the saturation coverage of precursors significantly deviates from the ideal closed packing. This mechanism, referred to as random sequential adsorption (RSA), is important for the understanding of the ALD processes.

RSA of different shapes/substances has been a topic of interest in different fields from physical chemistry and biology to the car parking problem.^23−27^ In order to study these various phenomena, different types of RSA simulation methods were developed considering continuum, or lattice-based approaches in one-dimensional, two-dimensional, and three-dimensional models.^28^ More specifically, the two-dimensional lattice RSA simulations have been used for the investigation of the packing of small molecule inhibitors (SMIs) during area-selective ALD.^19,20,29,30^ By enabling the evaluation of the SMI packing and the precursor-blocking performance of the SMIs, these applications showed the potential of RSA simulations for the ALD field.

In this work, we investigate the effect of RSA on the adsorption and coverage of the precursor molecules on the surface and thereby on the growth rate of the ALD film. By studying the outcomes of RSA simulations, the effect of precursor size and shape on the adsorption density is investigated. This information is used to calculate the ML coverage after 1 ALD cycle, i.e., ratio of the number of precursor molecules adsorbed and the number of metal atoms in a closed ALD layer. ML coverage is then used to estimate the GPC of the ALD process. Obtained results are reported in the following order: (i) comparison of theoretical ML coverages for ordered adsorption and RSA by using precursors with cyclopentadienyl (Cp) ligands and (ii) studying the influence of the precursor size on the GPC of an ALD process and the comparison of estimated GPC with experimental GPC values.

Two-dimensional lattice RSA simulations were used throughout this work. As the name implies, the two-dimensional substrate used for this type of RSA simulations is created based on the parameters of the crystalline lattice of the target ALD surface.^20,31^ The RSA simulations in this manuscript are formulated to use only the simplest physical properties of the precursor molecules and the substrate, i.e., molecular size and lattice dimensions. Therefore, other than the most probable adsorption geometry of the precursor, more complex mechanisms, such as cooperative effects, diffusion, or desorption of the adsorbates, were not considered in the current RSA model. For detailed discussion on the assumptions and limitations of RSA methodology, please see our previous work (ref (20)). In all cases, a substrate containing five thousand metal atoms was considered with periodic boundary conditions. Lattice dimensions of the surface and the position of the adsorption sites were adopted from density functional theory (DFT) optimized geometries. Here, we consider a perfect crystalline structure of the thermodynamically most favorable surface termination. RSA simulations were performed based on the in-house RSA script used in our previous work.^19,20^ Adsorption of the precursor molecules, represented by 2D footprints of their adsorbed geometry, on the substrate is studied by selecting random surface sites in every iteration. If an overlap with another molecule was detected, then the molecule was rotated and attempted to be placed again. If none of the possible random rotations resulted in adsorption, then the surface site was flagged, and a new site was chosen. The simulations were terminated after all adsorption sites on the surface were tested. The detailed algorithm of RSA simulations is explained in the computational method section in the Supporting Information.

We considered homoleptic precursor molecules with two ligands of the same kind. It is assumed that the selected precursors have a single adsorption configuration on the substrate by removal of one of its ligands. Therefore, extensive thermodynamic studies of the activity of different ligands on the substrate are not needed. In some cases, the ligand removed from the precursor can also adsorb on the surface, lowering the GPC of the process. However, this phenomenon needs to be further studied via either experiments or DFT simulations. Without these data, we assume that in all cases, the removed ligand will form a volatile product and desorb, without compromising neighboring surface sites. To eliminate geometric variations due to the nature of the substrate, we considered only ALD layers with a hexagonal surface lattice. To keep the precursor size versus lattice size comparison as simple as possible, only the precursors that can be represented with a symmetric 2D footprint geometry on the adsorption sites are considered. By screening the AtomicLimits ALD database for the presence of reported GPC data,^32^ we selected four different types of precursors namely metallocene (M(Cp)_2_), metal(II) acetylacetonate (M(acac)_2_), metal(II) hexafluoroacetylacetonate (M(hfac)_2_), and metal(II)bis(2,2,6,6-tetramethyl-3,5-heptanedionato) (M(thd)_2_). The 2D representations of the selected precursor models (after adsorption to the substrate) are visualized in Figure 1. This selection of precursors enabled the analysis of the results in two distinct groups. First, the precursors with Cp ligands can easily be represented as circles in the RSA simulations. This shape does not require any rotation during RSA simulations; therefore, the results obtained for the precursors with Cp ligands are purely dependent on the ratio between the radius of the Cp ligand and the lattice constant. Second, precursors with acac, hfac, and thd ligands all result in similar discorectangular 2D footprints with different sizes. This group serves as a direct comparison of the size effect of the precursor for the deposition of the same material. As an additional demonstration of the potential of this approach, we also present the comparison of an extended set of RSA-estimated GPC values with the experimental findings from the AtomicLimits ALD database.^32^

**Figure 1:**
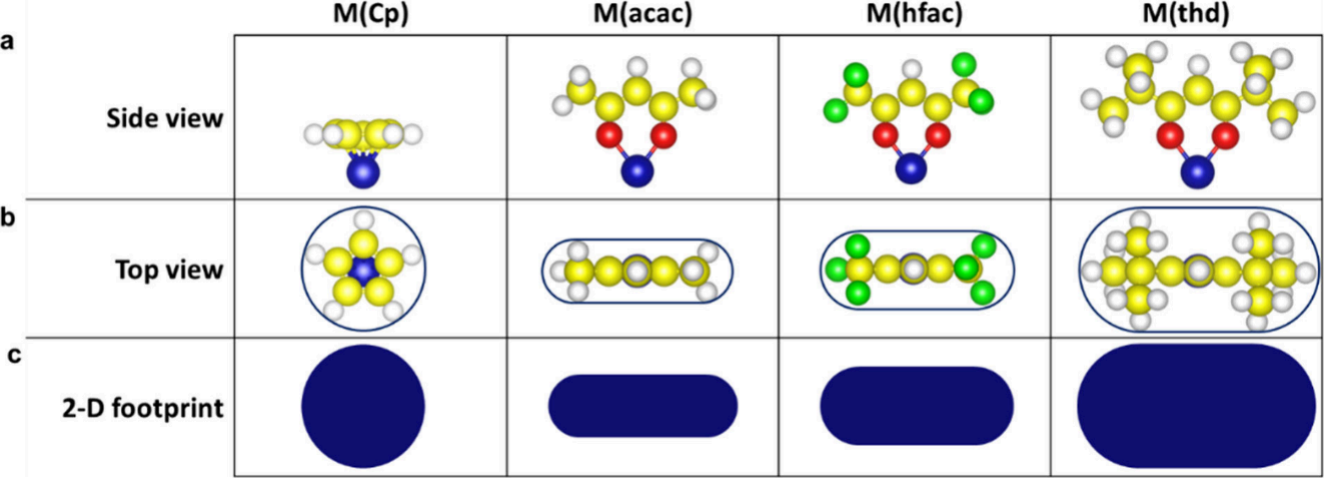
(a) Side and (b) top views of the adsorbed model precursor molecules visualized based on their van der Waals radius, and (c) the 2D footprints used in the RSA simulations following the same projection approach used in our previous work.^20^

As the first step of our work, we show the different outcomes of the random and ordered adsorption of metallocene precursor molecules on the ML coverage achieved during an ALD process. To represent the adsorbed precursor molecule as accurately as possible in the RSA simulations, DFT calculations were performed to study the adsorption of a commonly used metallocene precursor, cobaltocene, Co(Cp)_2_, on the Co (0001) surface. DFT calculations were carried out using the same parameters as in our previous work on Co (0001) surfaces^19^ and are explained in detail in the computational methods section of the Supporting Information. As explained in the literature, during the precursor half-cycle of ALD, the M(Cp)_2_ precursor loses one of its Cp ligands and adsorbs on the surface as MCp.^33^ Therefore, our DFT calculations are focused on the identification of the energetically favorable adsorption configuration(s) of this MCp structure on the surface. As shown by the orange labels in Figure 2, the hexagonal Co (0001) surface has four different adsorption sites available for the incoming precursor molecules. During DFT simulations, CoCp favorably adsorbs on *hcp* or *fcc* hollow sites without preference as the difference in total energies is less than 0.1 eV. DFT simulations starting on the top and bridge sites were converged to one of the hollow sites. The DFT results fulfilled the expectation of single adsorption configuration of the precursor molecule, in this case on hollow sites. Accordingly, RSA simulations were performed based on the precursor adsorption on the hollow sites of a hexagonal 2D grid.

**Figure 2:**
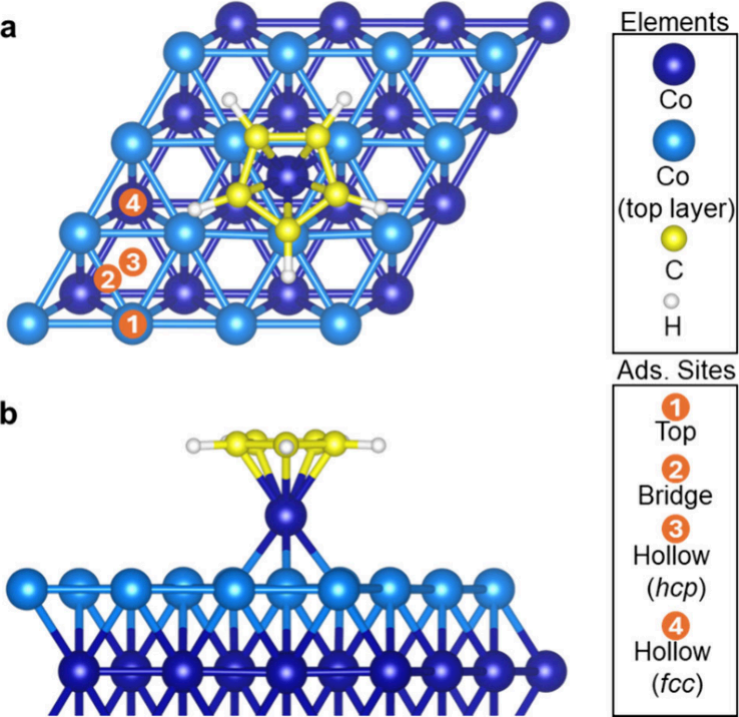
(a) Top and (b) side views of the optimized adsorption geometry of the cobaltocene (Co(Cp)_2_) precursor after losing one of its ligands. Among four different adsorption sites on the surface, a Co(Cp)_2_ precursor molecule prefers to adsorb on the hollow (either *hcp* or *fcc*) sites (see Figure S1). The top layer Co atoms of the Co (0001) surface have been colored differently to enhance the visibility. The legend on the right shows the different elements and adsorption sites.

Using the top view of the DFT output and the van der Waals radius of the elements, the 2D footprint of the metallocene molecules was considered in RSA simulations as a circle with a radius of 3.51 Å. In order to have an extensive data set, metallocene molecules with hypothetical metal centers have been considered such that ALD layers with various lattice constants, *a*, can be simulated. The van der Waals radius of potential metal centers^34^ for these hypothetical precursor molecules are always smaller than the radius of the Cp ligand, such that the dimensions of the 2D footprint are solely determined by the Cp ligand. Therefore, all hypothetical metallocene molecules are represented with the same radius, *r*, for the screening. A ML coverage data set is generated based on hexagonal substrates and circular precursor footprints as summarized in Figure 3a. We have updated our previous RSA model^20^ to consider hollow sites instead of top sites on the surface (see the Supporting Information). The reference simulations for the ordered adsorption are performed by testing the next adsorption site in the *y*-direction using a similar algorithm instead of randomly selecting an adsorption site. As shown in Figure 3a, full ML coverage is only possible when the surface lattice, *a*, is larger than twice the value of the precursor radius, *r*, which is in practice not possible. For such large lattice constants, it does not matter whether the adsorption is ordered or random, as shown in the first column of Figure 3b, since both ordered and random adsorptions result in the same coverage of 1 ML. As the *r*/*a* ratio increases, the ML coverage sharply decreases because of steric limitations. Due to the size of the molecules, adsorption on the nearest neighbor sites becomes unfavorable. Between the *r*/*a* ratios of 0.5 and 1.0, the ordered and random sequential adsorption simulations result in a 40% difference in the ML coverage. For CoCp_2_ on the Co (0001) surface, for example, *r*/*a* is 1.42, for which ordered and random sequential adsorption simulations calculate the ML coverage with a 22% difference. As can be seen in Figure 3, for more realistic cases, where the radius of the precursor molecule is larger than half of the lattice (*r*/*a* > 0.5), the random adsorption always results in a lower ML coverage than ordered adsorption.

**Figure 3:**
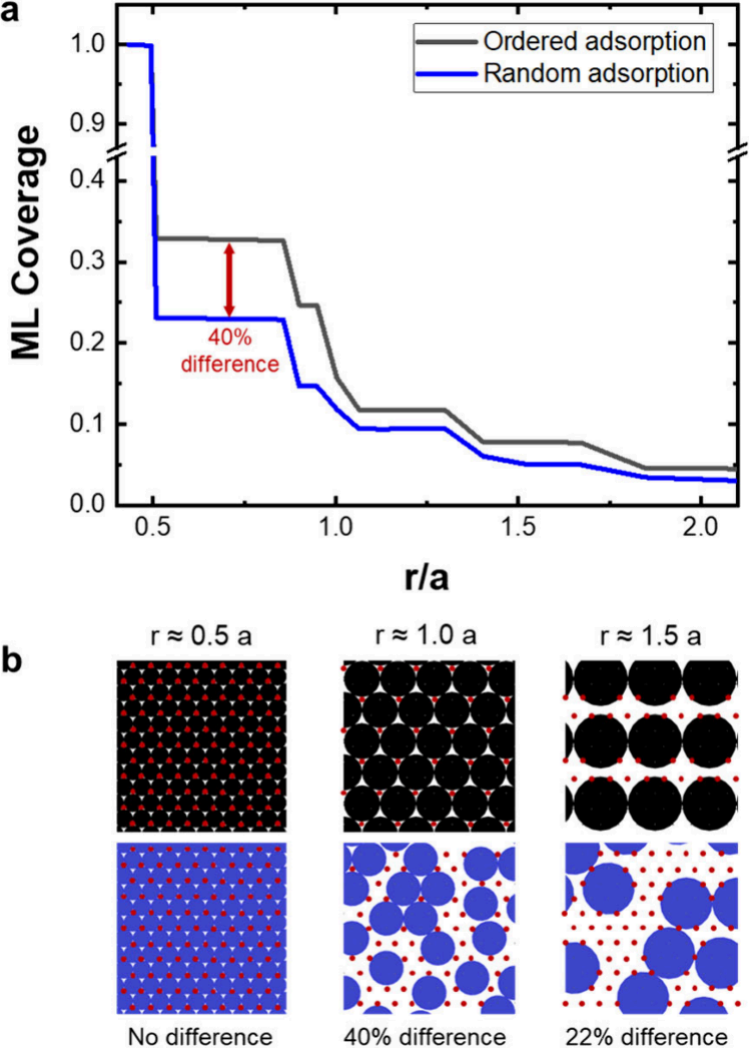
(a) Monolayer (ML) coverage as a function of the ratio of the precursor radius (*r*) to surface lattice constant (*a*) when a M(Cp)_2_ precursor with a circular 2D footprint is adsorbed on a hexagonal surface grid. (b) The visuals obtained from the ordered (top row) and random (bottom row) sequential adsorption of molecules with various *r*/*a* ratios. Red dots represent unoccupied adsorption sites after saturation.

The size of the precursor molecules has a significant effect on the growth rate of the ALD processes. To explain this effect, we studied Cu ALD using three different precursors with β-diketonate ligands of different sizes: copper(II) acetylacetonate (Cu(acac)_2_), copper(II) hexafluoroacetylacetonate (Cu(hfac)_2_), and copper(II)bis(2,2,6,6-tetramethyl-3,5-heptanedionato) (Cu(thd)_2_). During the precursor half-cycle of the Cu ALD process, Cu(acac) prefers to adsorb on the hollow sites of the Cu(111) surface, according to our DFT simulations (see Figure S1). Similarly, Cu(hfac) and Cu(thd) are expected to adsorb on the hollow sites. For all three precursors, the 2D footprint of the resulting adsorbate has a discorectangular shape, as shown in Figure 1. Once the ML coverage of the precursor on the surface is calculated using RSA simulations, the GPC can be estimated by multiplying the ML coverage and the theoretical thickness of one ML of the deposited material obtained from DFT (see Table S1 for details). Figure 4 shows the comparison between calculated GPC values and the values from the experimental literature. Here, also in line with the experimental findings from the literature, the inverse relation between the precursor size and the GPC is clearly visible, such that the Cu(acac)_2_ precursor which has the smallest 2D footprint leads to a higher Cu GPC compared to the larger Cu(hfac)_2_ and Cu(thd)_2_ precursors.^35−37^ Due to its smaller size, an acac ligand occupies a smaller space on the surface than the larger hfac and thd ligands, resulting in more Cu(acac)_2_ precursors on the surface, corresponding to a higher GPC. As the sizes of acac and hfac ligands are similar to each other, there is less difference in the GPC of Cu(acac)_2_ and Cu(hfac)_2_ precursors. Random and ordered adsorption simulations of the Cu precursors have resulted in coverages of 0.13 and 0.18 ML for acac, 0.11 and 0.15 ML for hfac, and 0.07 and 0.09 ML for thd ligands, respectively. The GPC values estimated via RSA and ordered simulations show on average, 0.05 and 0.12 Å deviations from the experimental values as shown in Figure 4, respectively. However, both RSA and ordered adsorption simulations showed a GPC similar trend as the precursor size changes.

**Figure 4:**
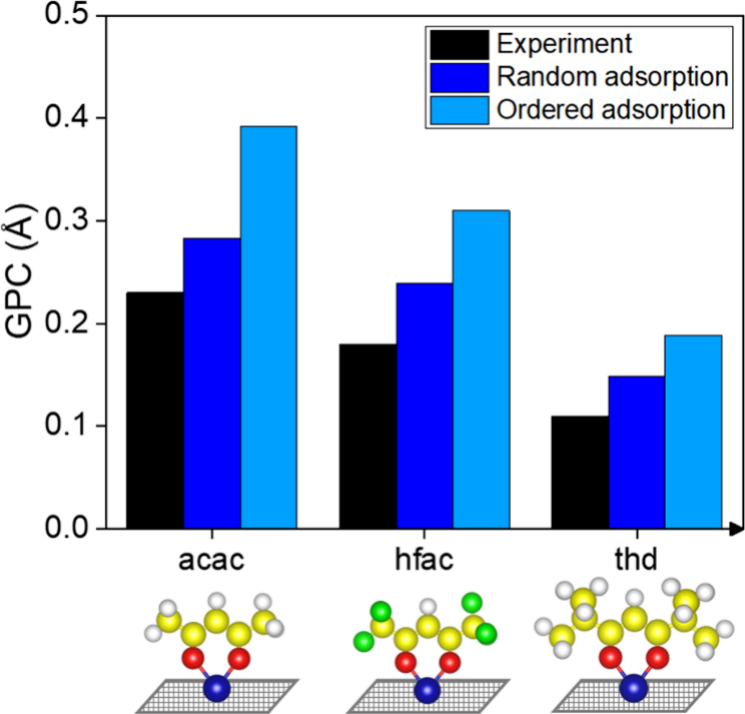
Comparison of experimental GPC values of Cu ALD with the GPC values calculated from random sequential adsorption and ordered adsorption of the Cu precursors with acac, hfac, and thd ligands. Experimental GPC data from references (35−37) are used in this comparison.

The above examples show that the GPC of an ALD precursor can be estimated based on its two-dimensional footprint using RSA simulations. In Figure 5, our experimental data set is expanded with more processes using the homoleptic precursor molecules with Cp, acac, hfac, and thd ligands included in the AtomicLimits ALD database.^32^ When collecting GPC data, plasma-assisted ALD processes are preferred, since these processes yield fewer complications due to partial ligand removal during the coreactant step.

**Figure 5:**
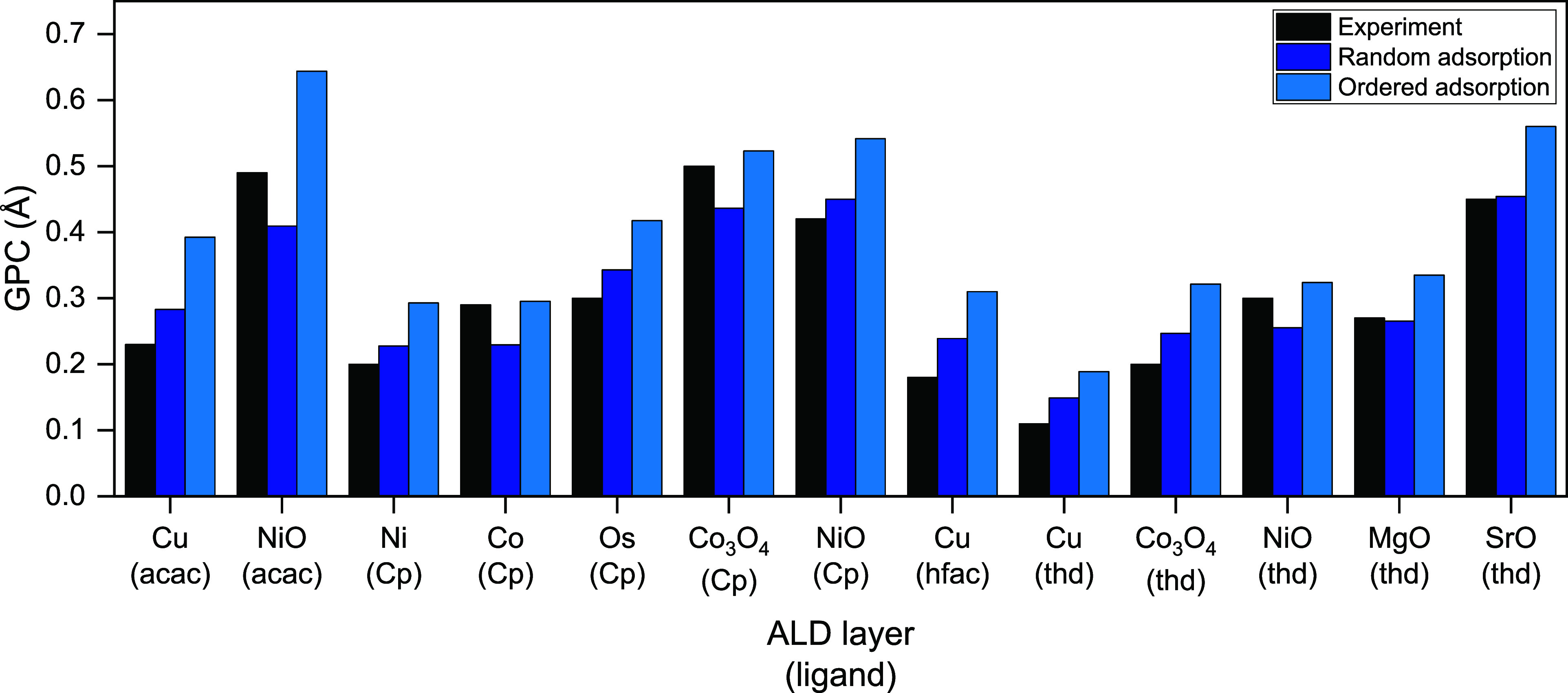
Comparison of experimental GPC and calculated GPC for the extended data set. The type of ligand used is stated in parentheses below the material. Experimental data is obtained from references (33, 35−37, 39−47). Please see Table S1 for more detailed data.

Figure 5 shows that in all cases, ordered adsorption overstimates the GPC in comparison to random adsorption. The comparison of the estimated GPC data with the experimentally reported values results in a mean absolute error of 0.05 Å for RSA and 0.10 Å for ordered adsorption. For the NiO process using acac and thd ligands and for the Co, and Co_3_O_4_ processes with Cp ligand, RSA simulations return GPC values lower than the experimentally observed values. The main reason for this difference on the oxide surfaces is attributed to the amorphous nature of the oxide surfaces in experimental conditions, which changes the distribution of surface sites. For example, Koushik et al. reported the presence of (111), (200), (220), and (311) planes in their NiO thin films.^38^ Different planes within the same substrate exhibit variations in their geometrical structures, reactive sites for adsorption, and interlayer distances, which can lead to varying average GPC values. Only the most densely packed (thermodynamically most favorable) planes of the materials are used to estimate the GPC in this work. The underestimated RSA-calculated GPC in Figure 5 signals the polycrystalline or amorphous nature of the experimentally deposited ALD layer. As seen in Figure 5, all metal ALD processes follow the same GPC trend between experiments and estimations, except for Co ALD with Cp ligands. Since the lattice dimensions and the monolayer thickness of Co and Ni surfaces are quite similar, the estimated GPC values obtained from random adsorption and ordered adsorption simulations are the same for ALD of these layers with Cp precursor. However, experimental GPC of the Co(Cp) process is significantly higher than the Ni ALD using Ni(Cp)_2_ precursor. Therefore, for Co ALD using Co(Cp)_2_ precursor, we suspect that there are discrepancies with experiments (e.g., such as chemical vapor deposition type-reactions), resulting in a higher GPC. Therefore, RSA simulations can both deliver an accurate estimation of the GPC of the process and also give information about the potential variations in the experimentally deposited layer.

As a summary, the understanding of random sequential adsorption of precursor molecules is essential to evaluate the performance of a precursor molecule during the ALD process. Due to the gas-phase nature of ALD processes, precursor molecules arrive on random surface sites one by one, and therefore, closed packing cannot be achieved. We showed that a surface can accommodate up to 40% fewer molecules when molecules are adsorbed according to the random sequential adsorption mechanism as compared to closed packing. Hence, recognizing the random nature for the adsorption of gas phase molecules has been proven to be key to understanding ALD growth. Following the methodology in this work, RSA simulations can be used to gain insights into the GPC values of precursors for specific ALD processes within an accuracy of ±0.05 Å.

## Supporting Information Available

Computational methods, RSA algorithm, tabulated experimental and estimated GPC data (PDF)
