# Supplementary material for: The Consequences of Random Sequential Adsorption for the Precursor Packing and Growth-Per-Cycle of Atomic Layer Deposition Processes
Source: J Phys Chem Lett. 2024 Jul 16;15(29):7496–501. doi: 10.1021/acs.jpclett.4c01632 (PMC11284847; doi:10.1021/acs.jpclett.4c01632)
Supplement: Supplementary file 1 — jz4c01632_si_001.pdf [file jz4c01632_si_001.pdf]

Supplementary Information:

The Consequences of Random Sequential Adsorption for the Precursor Packing and Growth-Per-Cycle of Atomic Layer Deposition Processes

I. Tezsevin,[a] J. H. Deijkers,[a] M. J. M. Merkx,[a] W. M. M. Kessels,[a] T.E. Sandoval,[b+] A. J. M. Mackus[a+]

[a] *Department of Applied Physics and Science Education, Eindhoven University of Technology, P.O. Box 513, 5600 MB Eindhoven, The Netherlands*

[b] *Department of Chemical and Environmental Engineering, Universidad Técnica Federico Santa Mariá, 2340000, Santiago, Chile*

[+] Corresponding author: a.j.m.mackus@tue.nl, tania.sandoval@usm.cl

COMPUTATIONAL METHODS

Density Functional Theory Calculations

The Vienna *ab-initio* Simulation Package (VASP) was used to perform all density functional theory (DFT) calculations reported in this study.[1–3] The projector augmented wave (PAW) method was employed to describe electron-ion interactions.[4,5] A kinetic energy cutoff of 400 eV was used for the plane-wave basis set. Calculations were performed based on Perdew-Burke-Ernzerhof (PBE) exchange-correlation functional of the generalized gradient approximation (GGA), with the dispersion correction D3 and the Becke–Johnson (BJ) damping function.[6–8] The convergence criteria for structural optimizations were set such that the total forces acting on each atom must be smaller than 0.01 eV/Å. Convergence criteria for the self-consistent-field cycle was set to 10^{-5} eV. The Brillouin zone of crystalline Co and Cu metal bulk was integrated using an automatically generated Γ centered 11 × 11 × 11 k-point mesh whereas a Γ centered 2 × 2 × 1 mesh was used for the surface calculations.[9] Gaussian smearing of 0.01 eV was used throughout the study. All calculations including Co system were performed using spin polarization.

Using these parameters, optimized lattice parameters of the Co bulk with P63/mmc space group (space group number= 194) were found to be a = 2.46 Å and c = 3.99 Å, which are in a good agreement with the experimental values of 2.50 Å and 4.06 Å, respectively.[10] The Co (0001) surface slab used for the surface calculations was modelled using a four layer 4 × 4 supercell of the cleaved optimized bulk structure.

The metal atoms in the bottom two layers of the surface slab were kept frozen at their bulk positions during adsorption studies. The periodicity of the slab in the direction perpendicular to the metal surface was avoided by adding a vacuum spacing of 17 Å. The thermodynamically most favorable adsorption geometries of CoCp on *fcc* and *hcp* hollow sites of the Co(0001) surface are reported in Figure S1.a-b.

Optimized lattice parameters of the Cu bulk with Fm3m space group (space group number= 225) was found as 3.56 Å, which is also in a good agreement with the literature (3.61 Å).[11] The methodologies used for the Co(0001) slab model and adsorption study are also followed for the modelling of Cu(111) surface slab and the Cu(acac) adsorbate. The thermodynamically most favorable adsorption geometries of Cu(acac) on *fcc* and *hcp* hollow sites of the Cu(111) surface are reported in Figure S1.c-d.

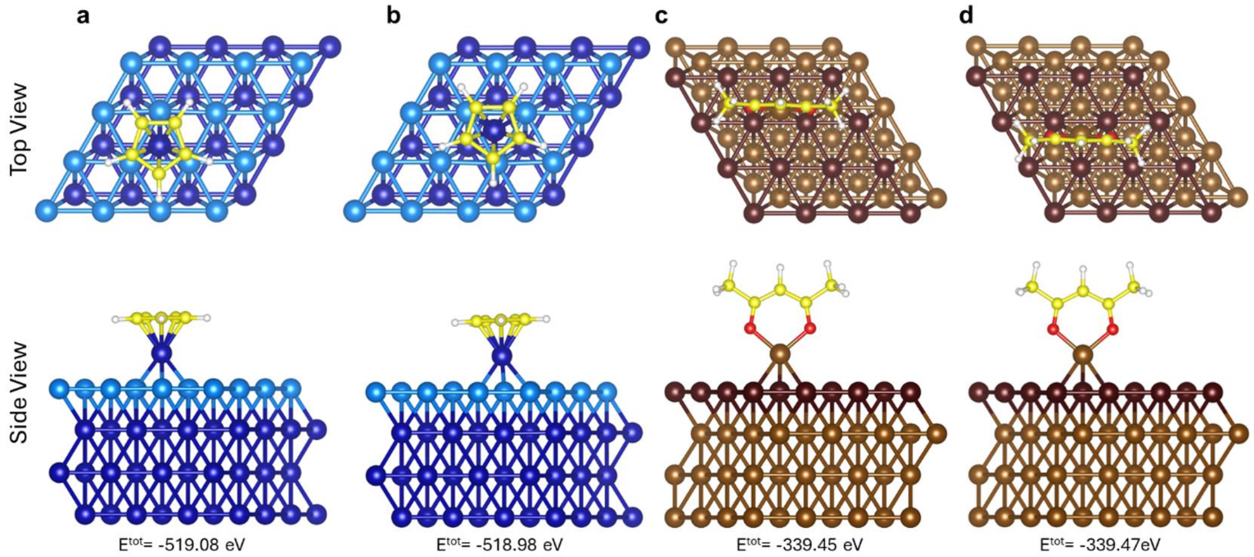

Figure S1. DFT optimized geometries of a-b) CoCp on a Co(0001) surface, and c-d) Cu(acac) on a Cu(111) surface. Top layer metal atoms of the Co (0001) and Cu (111) surfaces were colored differently to enhance visibility. Yellow: C, white: H, red: O, light/dark blue: Co, light/dark brown: Cu. Total energies of the optimized systems are reported below each configuration. Since the adsorption mechanism is not studied in detail, adsorption energies were not calculated.

Random Sequential Adsorption Simulations:

A lattice RSA algorithm (see Figure S2) developed based on our earlier work has been adopted in this study for the adsorption of the molecules on the hollow sites on the substrate (Please see *J. Vac. Sci. Technol. A* 2022, *40* (6), 062409 for more information on the RSA methodology.).[12] This RSA algorithm simulates one-by-one adsorption of precursor molecules on randomly selected sites on a substrate during the ALD process. The model substrates for all cases were prepared to include 5000 adsorption sites. The top-down 2D projections, i.e., 2D-footprints, of the precursor molecules were modelled based on the DFT-optimized adsorption configurations of molecules on the substrate (see Figure 1).

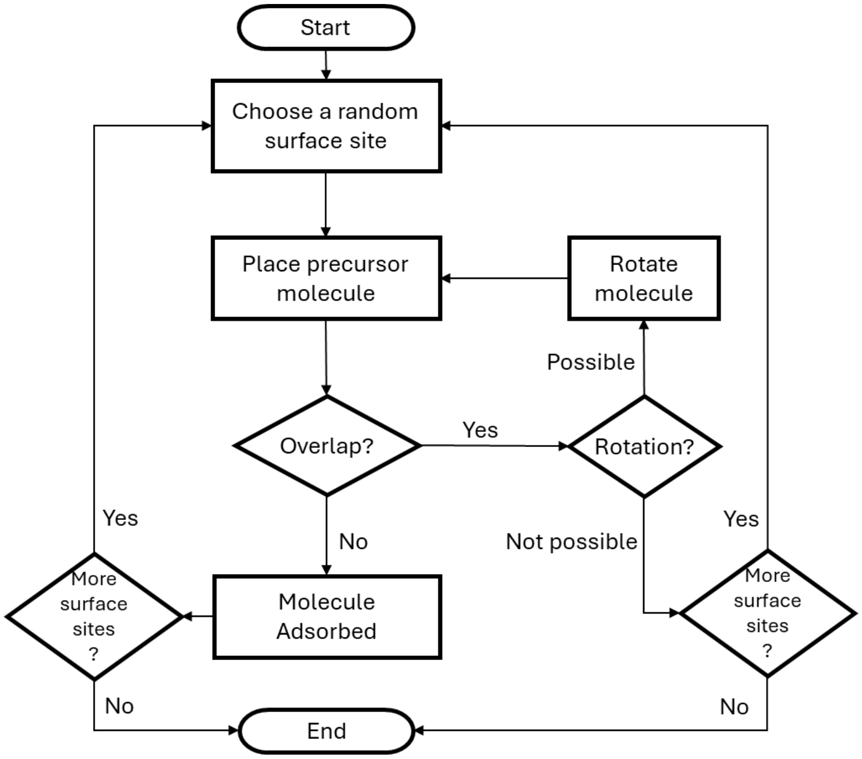

Figure S2. Algorithm followed in random sequential adsorption simulations.

In this algorithm, every surface site can be chosen only once in a random order. A molecule can only adsorb if there is no physical overlap with other molecules on the surface. After adsorption, molecules were frozen in that position until the end of the simulation. If an overlap with another molecule was detected, the molecule was rotated and attempted to be placed again. Due to the geometric properties, there is no rotation needed for the precursors represented with circular footprint. For the discorectangular shapes, a rotation step of 9° is applied. If none of the possible random rotations result in adsorption, the surface site was flagged, and a new site was chosen. The simulations were terminated after all adsorption sites on the surface were tested. Cooperative effects, diffusion, or desorption of the adsorbates were not

considered in the RSA model development. For the ordered adsorption simulations, the same algorithm is used except for the selection of the surface sites. During the ordered adsorption iterations, the next adsorption site is selected as the next site on y-direction by keeping the x component constant. When all sites are tested on the y-direction, new attempts are started from the beginning of the neighboring x component. All RSA results reported in this study were averaged over 10 simulations for the same process.

EXPERIMENTAL DATA AND ESTIMATION OF GPC

Experimental literature data and our RSA results used for the comparison in Figure 5 in the manuscript are reported in Table S1. Experimental references used in Table S1 sometimes report a GPC range as a function of different experimental conditions (e.g. temperature, dose time, etc.) . In these cases, the maximum GPC reported is adopted in the last column of Table S1 assuming better precursor adsorption kinetics without CVD component.

Table S1: Curated data used (columns 1-5) and obtained (columns 6-9) for the preparation of Figure 5. All precursor molecules reported in the table are represented with the formula M(ligand)$_2$ where the metal atoms and attached ligands are reported in columns 1 and 2. All surfaces reported in column 3 are modelled as hexagonal lattice in RSA simulations using the M-M distances reported in column 4. M-M distances and monolayer thickness values are obtained from DFT optimized perfect crystal structures. GPC is calculated as: $GPC^{RSA} = MLThickness^{DFT} \times MLcoverage^{RSA}$

Metal (M)	Ligand	ALD Surface	Surface M-M distance [Å]	Monolayer thickness [Å]	RSA Molecule Count	RSA Monolayer Coverage	RSA estimated GPC [Å]	Experimental GPC [Å]
Cu	acac	Cu	2.56	2.22	638	0.13	0.28	0.23 [13]
Ni	acac	NiO	2.98	2.98	678	0.14	0.40	0.49 [14]
Ni	Cp	Ni	2.48	2.15	530	0.11	0.23	0.20 [15]
Co	Cp	Co	2.46	2.13	531	0.11	0.23	0.29 [16]
Os	Cp	Os	2.73	2.36	724	0.14	0.34	0.30 [17]
Co	Cp	Co_3O_4	2.97	2.97	725	0.15	0.43	0.50 [18]
Ni	Cp	NiO	2.98	2.98	745	0.15	0.44	0.42 [19]
Cu	hfac	Cu	2.56	2.22	539	0.11	0.24	0.18 [20]
Cu	thd	Cu	2.56	2.22	336	0.07	0.15	0.11 [21]
Co	thd	Co_3O_4	2.97	2.97	410	0.08	0.24	0.20 [22]
Ni	thd	NiO	2.98	2.98	423	0.08	0.25	0.30 [23]
Mg	thd	MgO	3.01	3.01	435	0.09	0.26	0.27 [24]
Sr	thd	SrO	3.68	3.68	609	0.12	0.45	0.45 [25]

REFERENCES

(1) Kresse, G.; Furthmüller, J. Efficiency of Ab-Initio Total Energy Calculations for Metals and Semiconductors Using a Plane-Wave Basis Set. *Comput. Mater. Sci.* **1996**, *6* (1), 15–50. https://doi.org/10.1016/0927-0256(96)00008-0.

(2) Kresse, G.; Furthmüller, J. Efficient Iterative Schemes for Ab Initio Total-Energy Calculations Using a Plane-Wave Basis Set. *Phys. Rev. B - Condens. Matter Mater. Phys.* **1996**, *54* (16), 11169–11186. https://doi.org/10.1103/PhysRevB.54.11169.

(3) Kresse, G.; Hafner, J. Ab Initio Molecular Dynamics for Open-Shell Transition Metals. *Phys. Rev. B* **1993**, *48* (17), 13115–13118. https://doi.org/10.1103/PhysRevB.48.13115.

(4) Blöchl, P. E. Projector Augmented-Wave Method. *Phys. Rev. B* **1994**, *50* (24), 17953–17979. https://doi.org/10.1103/PhysRevB.50.17953.

(5) Joubert, D. From Ultrasoft Pseudopotentials to the Projector Augmented-Wave Method. *Phys. Rev. B - Condens. Matter Mater. Phys.* **1999**, *59* (3), 1758–1775. https://doi.org/10.1103/PhysRevB.59.1758.

(6) Perdew, J. P.; Burke, K.; Ernzerhof, M. Generalized Gradient Approximation Made Simple. *Phys. Rev. Lett.* **1996**, *77* (18), 3865–3868. https://doi.org/10.1103/PhysRevLett.77.3865.

(7) Grimme, S.; Antony, J.; Ehrlich, S.; Krieg, H. A Consistent and Accurate Ab Initio Parametrization of Density Functional Dispersion Correction (DFT-D) for the 94 Elements H-Pu. *J. Chem. Phys.* **2010**, *132* (15), 154104. https://doi.org/10.1063/1.3382344.

(8) Grimme, S.; Ehrlich, S.; Goerigk, L. Effect of the Damping Function in Dispersion Corrected Density Functional Theory. *J. Comput. Chem.* **2011**, *32* (7), 1456–1465. https://doi.org/10.1002/jcc.21759.

(9) Monkhorst, H. J.; Pack, J. D. Special Points for Brillouin-Zone Integrations. *Phys. Rev. B* **1976**, *13* (12), 5188–5192. https://doi.org/10.1103/PhysRevB.13.5188.

(10) Ono, F.; Maeta, H. Determination of Lattice Parameters in Hcp Cobalt By Using X-Ray Bond'S Method. *Le J. Phys. Colloq.* **1988**, *49* (C8), C8-63-C8-64. https://doi.org/10.1051/jphyscol:1988818.

(11) Straumanis, M. E.; Yu, L. S. Lattice Parameters, Densities, Expansion Coefficients and Perfection of Structure of Cu and of Cu–In α Phase. *Acta Crystallogr. Sect. A* **1969**, *25* (6), 676–682. https://doi.org/10.1107/S0567739469001549.

(12) Li, J.; Tezsevin, I.; Merkx, M. J. M.; Maas, J. F. W.; Kessels, W. M. M.; Sandoval, T. E.; Mackus, A. J. M. Packing of Inhibitor Molecules during Area-Selective Atomic Layer Deposition Studied Using Random Sequential Adsorption Simulations. *J. Vac. Sci. Technol. A* **2022**, *40* (6), 062409.

https://doi.org/10.1116/6.0002096.
(13) Wu, L.; Eisenbraun, E. Hydrogen Plasma-Enhanced Atomic Layer Deposition of Copper Thin Films. *J. Vac. Sci. Technol. B Microelectron. Nanom. Struct. Process. Meas. Phenom.* **2007**, *25* (6), 2581–2585. https://doi.org/10.1116/1.2779050.
(14) Attri, R.; Panda, D. P.; Ghatak, J.; Rao, C. N. R. High Crystalline Epitaxial Thin Films of NiO by Plasma-Enhanced ALD and Their Properties. *APL Mater.* **2023**, *11* (9). https://doi.org/10.1063/5.0157628.
(15) Wang, Y.-P.; Ding, Z.-J.; Liu, Q.-X.; Liu, W.-J.; Ding, S.-J.; Zhang, D. W. Plasma-Assisted Atomic Layer Deposition and Post-Annealing Enhancement of Low Resistivity and Oxygen-Free Nickel Nano-Films Using Nickelocene and Ammonia Precursors. *J. Mater. Chem. C* **2016**, *4* (47), 11059–11066. https://doi.org/10.1039/C6TC03606F.
(16) Vos, M. F. J.; Van Straaten, G.; Kessels, W. M. M. E.; Mackus, A. J. M. Atomic Layer Deposition of Cobalt Using H2-, N2-, and NH3-Based Plasmas: On the Role of the Co-Reactant. *J. Phys. Chem. C* **2018**, *122* (39), 22519–22529. https://doi.org/10.1021/acs.jpcc.8b06342.
(17) Hämäläinen, J.; Sajavaara, T.; Puukilainen, E.; Ritala, M.; Leskelä, M. Atomic Layer Deposition of Osmium. *Chem. Mater.* **2012**, *24* (1), 55–60. https://doi.org/10.1021/cm201795s.
(18) Donders, M. E.; Knoops, H. C. M.; Van, M. C. M.; Kessels, W. M. M.; Notten, P. H. L. Remote Plasma Atomic Layer Deposition of Co3O4 Thin Films. *J. Electrochem. Soc.* **2011**, *158* (4), G92. https://doi.org/10.1149/1.3552616.
(19) Hufnagel, A. G.; Henß, A.; Hoffmann, R.; Zeman, O. E. O.; Häringer, S.; Fattakhova-Rohlfing, D.; Bein, T. Electron-Blocking and Oxygen Evolution Catalyst Layers by Plasma-Enhanced Atomic Layer Deposition of Nickel Oxide. *Adv. Mater. Interfaces* **2018**, *5* (16). https://doi.org/10.1002/admi.201701531.
(20) Kang, S.-W.; Yun, J.-Y.; Chang, Y. H. Growth of Cu Metal Films at Room Temperature Using Catalyzed Reactions. *Chem. Mater.* **2010**, *22* (5), 1607–1609. https://doi.org/10.1021/cm902294e.
(21) Jezewski, C.; Lanford, W. A.; Wiegand, C. J.; Singh, J. P.; Wang, P.-I.; Senkevich, J. J.; Lu, T.-M. Inductively Coupled Hydrogen Plasma-Assisted Cu ALD on Metallic and Dielectric Surfaces. *J. Electrochem. Soc.* **2005**, *152* (2), C60. https://doi.org/10.1149/1.1850340.
(22) Klepper, K. B.; Nilsen, O.; Fjellvåg, H. Growth of Thin Films of Co3O4 by Atomic Layer Deposition. *Thin Solid Films* **2007**, *515* (20–21), 7772–7781. https://doi.org/10.1016/j.tsf.2007.03.182.
(23) Lindahl, E.; Lu, J.; Ottosson, M.; Carlsson, J.-O. Epitaxial NiO (100) and NiO (111) Films Grown by Atomic Layer Deposition. *J. Cryst. Growth* **2009**, *311* (16), 4082–4088.

https://doi.org/10.1016/j.jcrysgro.2009.06.030.

(24) Putkonen, M.; Johansson, L.-S.; Rauhala, E.; Niinistö, L. Surface-Controlled Growth of Magnesium Oxide Thin Films by Atomic Layer Epitaxy. *J. Mater. Chem.* **1999**, *9* (10), 2449–2452. https://doi.org/10.1039/a904315b.

(25) Kosola, A.; Putkonen, M.; Johansson, L.-S.; Niinistö, L. Effect of Annealing in Processing of Strontium Titanate Thin Films by ALD. *Appl. Surf. Sci.* **2003**, *211* (1–4), 102–112. https://doi.org/10.1016/S0169-4332(03)00175-2.
